# Editorial: the 18th annual *Nucleic Acids Research* web server issue 2020

**DOI:** 10.1093/nar/gkaa528

**Published:** 2020-06-21

The 2020 version of *Nucleic Acids Research*'s web server issue marks a change. This 18th web server issue is the first one to be assembled without the editorial supervision of Gary Benson, who served as its executive editor for more than a decade and whose work has made the issue the most prominent resource for scientific web-based applications.

Along with Gary, two of the three website reviewers who helped us to examine and confirm whether web server issue proposals were ‘publication-ready’ stepped back. Thank you, Sean Corbett and David Jenkins, for the excellent work you did for the web server issue. I should not forget to thank Gary's wife, Fay Oppenheim, who helped him (and myself over the last year) by keeping all the proposals, authors and reviewers organized. It is hard to imagine how the web server issue would have been possible without her constant support.

And, last but not least, thank you Gary for inviting me as your successor. You left very large footprints and I will try my best to fill them.

Without Fay's help, the well-rehearsed historical system of sending proposals by email became impossible—she used to extract all the essentials such as author names, URLs, etc. from the mails, PDFs or Word documents and entered them into a database. All readers who have tried to write algorithms capable of reliably retrieving author data from unformatted text will know that this is not possible without curation, so I decided to set-up an upload portal for proposals instead (https://nar.bihealth.de/).

As in the past, proposals were first editorially checked for their suitability and afterwards, websites or stand-alone tools were tested before allowing the submission of a manuscript. Together with Martine Bernardes-Silva, *NAR*’s editorial manager, we decided to use Scholar One's system of manuscript invitations for the successful proposals. With this infrastructure, handling the 274 proposals we received this year became feasible.

One of the past website reviewers, Adam Simpkin, is still on board and I found three other reviewers. These are our current website examiners:

- Adam Simpkin holds a PhD in X-ray crystallography and is at the University of Liverpool, UK.
- Piotr Grabowski owns a PhD in computational systems biology and works for AstraZeneca in Cambridge, UK.
- Lennard Ostendorf is a medical doctor and a PhD student at the Charité – Universitätsmedizin Berlin, Germany.
- Sebastian Proft is a bioinformatician and PhD student in my group at the Berliner Institut für Gesundheitsforschung (Berlin Institute of Health), Germany.

Of course, the change of the team and the move to a new infrastructure caused some teething pains, so the proposal and website reviews took longer than before. I apologize for the delay and promise faster decisions for the next issue.

The biggest challenge for all of us was, however, the Covid-19 pandemic. My group's offices have been turned into a temporary intensive care unit and most labs were closed. Many reviewers were hence not able to deliver their reports in time and many authors had difficulties submitting their revisions. Nevertheless, we managed to finish this year's Issue in time, and I want to use the opportunity to thank everyone for their work despite the crisis.

However, closed labs, lost offices, or delayed publications are certainly a small sacrifice compared to the many people who lost their lives, family members or friends, their jobs or those who could not meet their loved ones for a long time.

This year's web server issue contains the following manuscripts:

**Table:** 

Short title	URL	Short description
**TeamTat**	https://www.teamtat.org	Collaborative text annotation
**SIBiLS**	http://candy.hesge.ch/SIBiLS	RESTful customizable search engine for biomedical literature
**PDBMD2CD**	https://pdbmd2cd.cryst.bbk.ac.uk/	Prediction of protein circular dichroism spectra
**AWSEM-Suite**	https://awsem.rice.edu/	Protein structure prediction based on coevolution and coarse-grained force field
**TopMatch-web**	https://topmatch.services.came.sbg.ac.at	3D matching of large assemblies of protein and nucleic acid chains
**ShiftCrypt**	http://bio2byte.be/shiftcrypt/	Biophysical alignment of proteins through NMR chemical shift values
**ARIAweb**	https://ariaweb.pasteur.fr	Automated NMR protein structure calculation
**ProteinsPlus**	https://proteins.plus	Interactive analysis of protein–ligand binding interfaces
**HomolWat**	http://lmc.uab.cat/homolwat/	Incorporation of ‘homologous’ water molecules into G protein-coupled receptor structures
**FATCAT 2.0**	http://fatcat.godziklab.org/	Comparison of the structural diversity of proteins
**Zebra2**	https://biokinet.belozersky.msu.ru/zebra2	Analysis of subfamily-specific and conserved positions in protein superfamilies
**TREND**	http://trend.zhulinlab.org/	Exploring protein function in prokaryotes
**PlaToLoCo**	http://platoloco.aei.polsl.pl/	Annotation of low complexity regions in proteins
**piNET**	http://pinet-server.org	Analysis and visualization of proteomics data
**WebPSN**	http://webpsn.hpc.unimore.it	Inference fingerprints of structural communication in biomacromolecules
**EnzymeMiner**	http://loschmidt.chemi.muni.cz/enzymeminer	Automated selection of putative enzymes
**Conserved Unique Peptide Patterns (CUPP)**	https://www.cupp.info	Functional annotation of carbohydrate active enzymes
**AlloSigMA 2**	http://allosigma.bii.a-star.edu.sg	Designing allosteric effectors and using allosteric effects of variants
**mmCSM-AB**	http://biosig.unimelb.edu.au/mmcsm_ab/	Guiding rational antibody engineering through multiple point mutations
**MISCAST**	http://miscast.broadinstitute.org/	Visualization and analysis of missense variants in protein sequences
**MusiteDeep**	https://www.musite.net	Post-translational modification site prediction and visualization
**mCSM-membrane**	http://biosig.unimelb.edu.au/mcsm_membrane/	Predicting the effects of variants on transmembrane proteins
**LIST-S2**	https://list-s2.msl.ubc.ca/	Prioritization of deleterious missense mutations in different species
**VarFish**	https://varfish-kiosk.bihealth.org	Collaborative exome analysis for clinic and research
**3D-GNOME**	http://3dgnome.cent.uw.edu.pl/	predicting structural variation-driven alterations of chromatin spatial structure in the human genome
**Galaxy HiCExplorer**	https://hicexplorer.usegalaxy.eu	Promoter capture Hi-C and single-cell Hi-C data analysis, quality control and visualization
**SNPnexus—2020 update**	https://www.snp-nexus.org/	Functional annotation of human genome sequence variation
**EpiRegio**	https://epiregio.de	Analysis and retrieval of regulatory elements linked to genes
**InteractomeSeq**	https://interactomeseq.ba.itb.cnr.it/	Identification and profiling of domains and epitopes from Phage Display and Next Generation Sequencing data
**TFmotifView**	http://bardet.u-strasbg.fr/tfmotifview/	Visualization of transcription factor motifs in genomic regions
**RiboToolkit**	http://rnabioinfor.tch.harvard.edu/RiboToolkit	Decoding RNA translation
**AnnoLnc**	http://annolnc.gao-lab.org	Annotation of novel lncRNAs in human and mouse
**mRNALoc**	http://proteininformatics.org/mkumar/mrnaloc	Predicting mRNA subcellular localisation
**miRNet 2.0**	https://www.mirnet.ca/	miRNA functional analysis
**miRViz**	http://mirviz.prabi.fr/	Visualization and interpretation of microRNA datasets
**mirnaQC**	https://arn.ugr.es/mirnaqc/	Comparative quality control of miRNA-seq data
**miRSwitch**	https://anathema.cs.uni-saarland.de/mirswitch/	miRNA arm switch event finder
**IRIS3**	https://bmbl.bmi.osumc.edu/iris3/	Inference of cell-type-specific regulons from single-cell RNA-seq
**MutaRNA**	http://rna.informatik.uni-freiburg.de/MutaRNA	Analysis and visualization of mutation-induced changes in RNA structure
**RNAProbe**	https://iimcb.genesilico.pl/shape/	Normalization and analysis of RNA structure probing data
**rMAPS2**	http://rmaps.cecsresearch.org/	RNA map analysis for alternative splicing regulation
**SPEED2**	https://speed2.sys-bio.net/	Inferring upstream pathway activity from differential gene expression
**FGviewer**	https://ccsmweb.uth.edu/FGviewer	Visualization of functional features of human fusion genes
**PseudoChecker**	http://pseudochecker.ciimar.up.pt/	Automated discovery of pseudogenes
**OligoMinerApp**	http://oligominerapp.ingm.org/	Design of genome-scale oligonucleotide *in situ* hybridization probes
**BE-FF**	http://danioffenlab.pythonanywhere.com/BE-FF	Prediction of synonymous corrections for CRISPR/Cas
**PaCRISPR**	http://pacrispr.erc.monash.edu/	prediction of anti-CRISPR proteins
**AcrFinder**	http://bcb.unl.edu/AcrFinder	Search for anti-CRISPR operons in prokaryotes and their viruses
**NanoSPC**	https://nanospc.mmmoxford.uk/	A nanopore metagenomic data processing pipeline
**CReSCENT**	http://crescent.cloud	Cancer single-cell transcriptomics data analysis
**The Omics Discovery REST interface**	https://www.omicsdi.org/ws/	Access, discovery and dissemination of omics datasets
**BIOMEX**	https://www.vibcancer.be/software-tools/biomex	Single cell omics data interpretation and visualization
**Galaxy Collections**	http://galaxyproject.org	2020 update of the Galaxy platform for collaborative biomedical analyses
**ASAP**	https://asap-beta.epfl.ch/	Single-cell omics analyses
**Oviz-Bio**	https://bio.oviz.org	Interactive cancer genomics data visualization
**Fluxer**	https://fluxer.umbc.edu	Computation and analysis of genome-scale metabolic flux networks
**NOREVA 2.0**	https://idrblab.org/noreva2020/	Normalization and evaluation of time-course and multi-class metabolomic data
**NetMHCpan-4.1 and NetMHCIIpan-4.0**	http://www.cbs.dtu.dk/services/NetMHCIIpan-4.0/	Predictions of MHC antigen presentation
**ToxicoDB**	http://toxicodb.ca/	Mining large-scale toxicogenomic datasets
**CVCDAP**	http://omics.bjcancer.org/cvcdap/	Platform for molecular and clinical analysis of cancer virtual cohorts
**Tox21BodyMap**	https://sandbox.ntp.niehs.nih.gov/bodymap/	Mapping chemical effects on the human body
**novoPathFinder**	http://design.rxnfinder.org/novopathfinder/	Designing novel pathways
**SynergyFinder 2.0**	https://synergyfinder.fimm.fi	Visual analytics of multi-drug combination synergies
**SYNERGxDB**	http://SYNERGxDB.ca/	Identification of synergistic drug combinations for precision oncology
**PaccMann**	https://ibm.biz/paccmann-aas	Explainable anticancer drug sensitivity prediction
**TIMER2.0**	http://timer.cistrome.org/	Analysis of tumor-infiltrating immune cells
**GeneTrail 3**	http://genetrail.bioinf.uni-sb.de	High-throughput enrichment analysis
**miEAA 2.0**	https://ccb-compute2.cs.uni-saarland.de/mieaa2	Gene-set enrichment including multi-species microRNA analysis
**LINbase**	http://linbase.org	Genome-based identification of prokaryotes
**The Quest for Orthologs Continuous Benchmark Service and Consensus Calls 2020**	https://orthology.benchmarkservice.org/	Benchmarking the identification of orthologs
**ARTS 2.0**	http://arts.ziemertlab.com	Antibiotic Resistant Target Seeker for comparative genome mining
**MetaPhOrs**	http://orthology.phylomedb.org/	Phylogeny-based inference of orthology and paralogy
**COVTree**	http://www.lcqb.upmc.fr/COVTree/	study co-evolution in overlapped sequences
**CoCoCoNet**	http://milton.cshl.edu/CoCoCoNet	Co-expression in different species
**MetaNets**	https://web.rniapps.net/metanets/	Inference of microbial correlation networks
**SuperCypPred**	http://insilico-cyp.charite.de/SuperCYPsPred/	Prediction of cytochrome activity
**InterPred**	https://sandbox.ntp.niehs.nih.gov/interferences/	Prediction of chemical autofluorescence and luminescence interference
**Atomic Charge Calculator II**	http://ncbr.muni.cz/ACC2	Calculation of partial atomic charges
**CausalMGM**	http://causalmgm.org	A causal discovery tool

We received 273 proposals for this year's Web Server Issue, including four duplicates. Of these 269 proposals, exactly 100 (37%) were allowed to submit a manuscript. One manuscript was never submitted and another one retracted. Seventy nine manuscripts were finally accepted after peer-review (29% acceptance rate of proposals, 81% acceptance rate of submitted manuscripts).

The main reasons for an early rejection of manuscripts were:

- Proposal of a new method or focus on a single dataset

The web server issue is not aimed at publishing new approaches, its primary role is to collect user-friendly (mainly web-based) interfaces for established tools or methods. Many of the proposals rejected either proposed new methods (such as machine learning-based classifiers) and/or were aimed at analysis/visualization of a single dataset, which is mainly relevant for the authors themselves.

- Lack of user-friendliness/lacking or insufficient documentation

Software published in the web server issue must be easy-to-use, and well-documented. We will not accept manuscripts if software or documentation do not fulfill these criteria.

- No benefit over existing approaches

Tools such as R Shiny make it easy for non-programmers to produce web-based applications. While we have published web servers based on Shiny in the past and will continue to do so, these must provide a significant benefit over using the underlying R package without a web server, e.g. by providing an easy-to-use input, a user-friendly output, and extended visualization options. If users retrieve results only as R data frames and must hence use R anyway to handle them, there is no advantage in having a web server.

With the advent of web servers able to handle complete exomes or even genomes of humans, there is another problem emerging: data security and privacy.

One reviewer who studied a web server in more detail than we did, found a relatively simple way to access data uploaded by other users. We are very grateful to you! This was of course a wake-up call for us to put more emphasis on security/privacy issues when testing websites. But it also questioned our previous policy of forbidding logins—these are of course safer than URLs including a private accession ID. This point was indeed raised by several referees and we have thus decided to allow logins if—and only if—personal data such as human WGS or WES data is transmitted. For such websites, we will also require SSL (https) encryption. To ensure free access, users must be able to obtain a login without a working email address.

**Figure 1.**
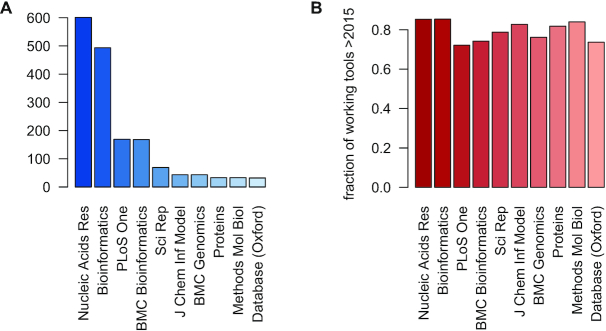
(**A**) Web services published since 2010, (**B**) Percentage of available web services published after 2015.

In the first paragraph I wrote that, thanks to Gary Benson's work, the *NAR* web server issue has become the most prominent resource for scientific web servers. This is illustrated in an unpublished study by Andreas Keller's group from Universität des Saarlandes, Saarbrücken, Germany. They extracted articles describing web services of all kind from PubMed and checked whether the services are still available. As shown in Figure 1A, *NAR* has published more web servers than any other journal in the last ten years and a high proportion of these are still running (Figure 1B). We are of course very proud of these numbers—thank you for sharing them. I hope that your study will resolve in a web-based database that will show which authors keep their web servers alive after publication.

With this encouraging study, I will close my first editorial. In addition to those mentioned above, I want to thank the other editors of *NAR* and the staff at Oxford University Press for their support, especially our publisher, Joanna Ventikos.

But most of all I am grateful to the authors and reviewers who did a remarkable job in these difficult times.

Please check https://academic.oup.com/nar/pages/submission_webserver for any changes of deadline or requirements by November if you want to submit to the 2021 web server issue.

